# Distribution of Environmental Phenols into Follicular Fluid and Urine of Women Attending Infertility Clinic

**DOI:** 10.3390/jox15010017

**Published:** 2025-01-21

**Authors:** Anna Klimowska, Joanna Jurewicz, Michał Radwan, Paweł Radwan, Paweł Pol, Bartosz Wielgomas

**Affiliations:** 1 Department of Toxicology, Faculty of Pharmacy, Medical University of Gdansk, 107 Hallera Street, 80-416 Gdańsk, Poland; anna.klimowska@gumed.edu.pl; 2 Department of Chemical Safety, Nofer Institute of Occupational Medicine, 91-348 Lodz, Poland; joanna.jurewicz@imp.lodz.pl; 3 Department of Gynecology and Reproduction, Gameta Hospital, 34/36 Rudzka St., 95-030 Rzgow, Poland; mradwan@gameta.pl; 4 Faculty of Health Sciences, Mazovian State University in Plock, 2 Dabrowskiego Sq., 09-402 Plock, Poland; 5 Gameta Health Centre, 7 Cybernetyki St., 02-677 Warsaw, Poland; pradwan@gameta.pl; 6 Gameta, Kielce-Regional Science-Technology Centre, 45 Podzamcze St., Chęciny, 26-060 Kielce, Poland; ppol@gameta.pl

**Keywords:** endocrine-disrupting compounds, female reproductive health, follicular fluid, urine

## Abstract

Infertility and environmental pollution are two globally prevalent and related issues. To explore women’s reproductive health, the composition of follicular fluid (FF) has been studied and it was found that changes to its composition, including the presence of exogenous chemicals, can adversely affect the fertilization process. Two groups of women (idiopathic infertility and controls) who were patients at a fertility clinic were recruited for this study. Samples of urine and FF were gathered from each participant to determine the concentration of 14 common phenols (four parabens, six bisphenols, two benzophenones, and two naphthols). Associations between phenol concentrations (free and total) in both matrices were described using Spearman’s correlation coefficient and were compared between two groups by the Mann–Whitney U test. Eight phenols were quantified in more than 50% of the urine samples, while only three parabens were quantified in hydrolyzed FF samples, and only methylparaben was quantified in non-hydrolyzed FF samples. Conjugates were the predominant form in FF samples. However, a significant correlation of 0.533 (*p* < 0.0001) was observed between free and total methylparaben concentrations in FF. Differences in concentrations between cases and controls in both matrices were not statistically significant, except for benzophenone-3 in urine, with a higher median observed in the control group (*p* = 0.04). The total paraben concentrations in urine and FF samples were rather weakly correlated (r = 0.232–0.473), implying that urine concentrations may not be appropriate for predicting their concentration in FF.

## 1. Introduction

Synthetic chemicals are an inseparable component of the modern world, often posing a hazard to human and animal health. The risk can arise from their widespread occurrence in the environment, but they are also intentionally added to many daily used consumer products or even food (e.g., parabens, bisphenols, or UV filters).

Several environmental pollutants are currently considered endocrine-disrupting compounds (EDCs) and are therefore being investigated by numerous researchers around the world. Imitating naturally occurring hormones, EDCs can influence the endocrine system, affecting the production, secretion, transport, or excretion of natural substances, as well as binding directly to the relevant receptors [1]. A number of biomonitoring studies have shown widespread exposure to EDCs among both women and men, but also children [2,3,4]. EDCs can modify sex or thyroid hormone levels, impair both female and male reproductive system function, reduce semen quality in men, or increase the time needed for pregnancy. The endocrine activity of EDCs has been reported in in vitro, animal, and human epidemiological studies. In the literature, data demonstrating the negative effects of EDCs have been summarized in several review papers [5,6,7,8]. It is worth noting that many synthetic EDCs feature a phenolic structure, which is linked to their endocrine-disrupting activity [1].

The assessment of exposure to EDCs is a complex process that requires consideration of many factors, including exposure to low doses of multi-substance mixtures over a prolonged amount of time. Hormones, by interacting with specific receptors, can manifest their activity at low concentrations, tending to a non-linear dose–response relationship. This characteristic is also shared by some compounds within the EDC group. Consequently, the effect can be maximized at both low and high doses (“U”-shaped dose–response curve) [1]. The combined effects of a natural hormone and EDCs may pose an additional risk [9]. Furthermore, the stage of human development at which exposure occurs is key. Exposure of pregnant woman to these can have a negative impact on both her and her offspring. More importantly, exposure during very early stages of development may not manifest itself until adolescence or even adulthood [10,11].

The follicular fluid (FF) forms the microenvironment for the development of the oocyte, which subsequently transforms into an ovum. The FF contains factors that are present in serum, such as hormones, enzymes, and fatty acids [12,13,14]. The development of the oocyte is strongly influenced by hormones (estrogen, follicle-stimulating hormone, or luteinizing hormone); accordingly, their optimal concentrations are crucial for proper growth and transformation into an ovum [15,16]. Xenobiotics in the FF, resultant from environmental exposure, are able to interrupt the action of natural hormones and adversely affect the quality of the oocyte, which may consequently hinder pregnancy or its delivery [17,18,19].

Data on the presence of EDCs in follicular fluid are still deficient and mainly target persistent organic pollutants (POPs), such as organochlorine compounds (OCs) [20,21], polybrominated diphenyl ethers (PBDEs) [18,19], or perfluoroalkyl substances (PFASs) [20,22]. On the presence of non-persistent EDCs in the FF, only phthalates [11,23,24] and some phenols, such as parabens or bisphenols [24,25,26,27], have been investigated. Aimed at expanding the knowledge on non-persistent EDCs, this study sought to (i) quantify the concentrations of 14 phenolic compounds in FF samples; (ii) characterize the relationship between the qualitative and quantitative profiles in another biological matrix (urine); (iii) compare measured concentrations between the group of women diagnosed with idiopathic infertility and the control group; and (iv) determine the form (free form or phase II metabolite) in which the studied substances occur in the FF.

## 2. Materials and Methods

### 2.1. Reagents and Materials

All the reagents, materials and analytical and internal standards are listed in the Supplementary Materials (Text S1).

### 2.2. Sample Collection

The study participants were women who attended a fertility clinic for diagnosis and treatment in 2018. Sixty-four women with idiopathic infertility were enrolled into the case group and ninety women in the control group (controls), for whom the cause of the couple’s infertility was on the male side. The distribution of age and socioeconomic status was comparable in both groups of women. The case and control group were aged 33.5 ± 3.7 yo and 33.2 ± 3.6 yo, respectively. The socioeconomic status was defined by educational level and all participants had a university degree. All women underwent a standard in vitro fertilization (IVF) procedure; one urine sample and follicular fluid were also collected as part of the procedure. Specimens were collected into polypropylene cups (urine) and tubes (FF) at stored at −80 °C after collection and, once transported to the laboratory of the Medical University of Gdańsk, at −20 °C until analysis.

This study was approved by the Independent Bioethics Commission for Research by the Medical University of Gdańsk (agreement No. NKBBN/24/2018). By signing a written consent form, all women validated their participation in this study.

### 2.3. Analytical Methods

Concentrations of four parabens (methyl (MP), ethyl (EP), propyl (PP), and butyl (BP) ester), six bisphenols (bisphenol A (BPA), bisphenol AF (BPAF), bisphenol B (BPB), bisphenol E (BPE), bisphenol F (BPF), and bisphenol S (BPS)), two benzophenones (benzophenone 1 (BP-1), and benzophenone 3 (BP-3), and two naphthols (1-naphthol (1-NP), 2-naphthol (2-NP)) in urine samples were analyzed using a validated method described elsewhere [28]. In that paper, it was also shown that phenolic compounds are excreted in urine mainly as conjugates; therefore, urine samples were analyzed only after hydrolysis. Thus, two milliliters of urine were treated with β-glucuronidase/sulfatase, heated overnight, and then acidified with formic acid. The hydrolysate of the urine sample was loaded onto a Bond Elut Plexa SPE column (Agilent, Santa Clara, CA, USA) previously conditioned with ethyl acetate, methanol and 1% formic acid in water. Prior to thorough drying, the sorbent was washed with 15% methanol in 1% formic acid in water, followed by elution with 2 × 250 µL of ethyl acetate. The dry residue was reconstituted with 50 µL of MSTFA and derivatized for 30 min at 60 °C. One microliter of the extract was injected into the GC-MS/MS system.

The method for the FF samples was developed based on the method described above for urine samples. One milliliter of FF was spiked with 20 µL of internal standard solution, diluted with an equal volume of water and then mixed with 250 µL of acetate buffer (pH 5.0). To measure the total concentration as a sum of the free and conjugated form, β-glucuronidase/sulfatase was added, followed by an overnight incubation at 37 °C. Prior to loading the sample, the sorbent was conditioned with 1 mL of dichloromethane, methanol, and water. Subsequently, it was washed with 2 × 1 mL of water and dried under vacuum for 30 min. Analytes were eluted in two fractions: (i) 2 × 500 µL of dichloromethane and (ii) 2 × 500 µL of 1% formic acid in ethyl acetate. Each fraction was collected separately, evaporated to near dryness under a gentle stream of nitrogen, and reconstituted with 50 µL of MSTFA. After derivatization (30 min, 60 °C), one microliter was injected into the GC-MS system. The same procedure, excluding enzymatic hydrolysis, was used to determine the free forms of the investigated biomarkers in FF samples.

Analyses were carried out employing the GC-MS/MS method previously described [28].

### 2.4. QA/QC

To ensure adequate purity of the materials used, all glassware was thoroughly washed beforehand and baked at 350 °C for four hours. In addition, to control the possibility of introducing contaminants during sample preparation, every batch of samples included procedural blanks, which did not contain the biological matrix, but did contain all reagents and materials used in the protocol. Only BPA was detected in the procedural blank, with an average concentration of 0.122 ng/mL.

Control samples (QC) were prepared in-house by spiking pooled urine or FF with all analytes of interest at 3–5 concentration levels (Tables S1 and S2). Forty real samples, one QC for every 10 real samples, and two procedural blanks were included in the daily batch. The mean concentration (accuracy) and inter-day precision were determined for all QC levels. The accuracy of 73–123% was obtained for FF, for all but 1-naphthol (<70%), and 71–115% for urine. The inter-day precision for both matrices was <30%.

For calibration curves, nine solutions were made using the serial dilution method and used to spike the blank urine and FF pools (matrix-matched calibration). A blank urine pool was prepared by mixing equal volumes of urine collected anonymously from six donors, while for the FF, 1 mL of each sample provided by study participants was taken and mixed as a pool. The LOD values were established as the lowest calibration point, which could be determined with a precision of 25%, and were 0.1–0.5 ng/mL for urine and 0.1–0.2 ng/mL for the FF (Tables S1 and S2). Linearity was observed in the range of LOD-600 ng/mL (r > 0.985) for urine and LOD-20 ng/mL (r > 0.990, except BPA r = 0.973) for the FF. Samples with concentrations measured above the highest point of the calibration curve were reanalyzed using lower volumes.

### 2.5. Statistical Analysis

The Shapiro–Wilk test was employed to confirm the normality of data distribution, revealing that the data did not follow a normal distribution; therefore, nonparametric tests were applied for subsequent analyses. The association between total concentrations in urine and FF samples was assessed using Spearman’s correlation coefficient, whereas the concentrations obtained for the case and control groups were compared employing the Mann–Whitney U test. The results were considered statistically significant when *p* < 0.05.

Concentrations < LOD were replaced by the LOD value divided by the root of two [29]. Only analytes with detection > 50% were subjected to the statistical analysis. All statistical analyses and graphs were produced in GraphPad Prism 5.0 (GraphPad Software, San Diego, CA, USA).

## 3. Results

### 3.1. FF Method Optimization

The follicular fluid is a complex matrix composed of multiple groups of substances, such as proteins and fatty acids, making the method development process challenging. Attempting direct liquid–liquid extraction with a mixture of MTBE/hexane (1:3; *v*:*v*) led to the formation of a stable gel, which prevented the collection of the organic layer and its further processing. On the other hand, the high viscosity of FF samples, compared, for example, to urine samples, and the presence of proteins precluded a smooth SPE extraction due to the slow sample flow through the sorbent or its clogging. Therefore, it was necessary to introduce a sample pretreatment step prior to extraction. The standard procedure for protein removal involves adding ACN in a 1:1 (*v*:*v*) ratio to the sample, which effectively precipitates the proteins, but the volume of ACN used required at least 10-fold dilution of the sample with water before extraction to achieve satisfactory recoveries for some analytes, such as MP or BPS. Since this project also aimed to separately measure the free and total concentration of biomarkers tested in the FF, each FF sample was analyzed twice: without and with enzymatic hydrolysis. Given the potential adverse impact of ACN on β-glucuronidase, especially at higher concentrations, and the large volume of the pretreated sample (10 mL), we decided to omit the use of ACN. Eventually, 1 mL of the FF was diluted with an equal volume of water, mixed with 0.25 mL of acetate buffer containing enzyme (pH 5.0) and incubated overnight. Enzymatic hydrolysis is usually stopped by adding formic acid; however, in the case of the FF samples, acid treatment led to sorbent clogging during extraction. Therefore, the reaction was stopped by placing the samples in an ultrasonic bath for 15 min.

A previously developed SPE protocol for urine samples (described in Section 2.3) was initially tested. However, clogging of the sorbent forced us to wash it after sample loading with water instead of the 10% methanol solution. Also, the elution step had to be re-optimized. The original protocol used ethyl acetate as a green solvent for elution, but it eluted interference with a retention time similar to BPA, disallowing the detection of BPA (Figure S1A). The analysis of the FF sample in total ion current mode (TIC) enabled a tentative identification of the contamination. A comparison of the obtained MS spectra with spectra available in the NIST library (NIST MS Search 2.0 Software, NIST, Gaithersburg, MD, USA) showed that the contaminants are likely fatty acids (Figure S2). Given their high lipophilicity, a two-step elution using dichloromethane (DCM) and then ethyl acetate (EA) was applied. Both fractions were analyzed separately in TIC mode. As a result, contamination was observed only in the DCM fraction (Figure S1A,B1). Nevertheless, a very low recovery (<1%) was observed for BPS in the EA fraction (Figure S3). Therefore, formic acid at a concentration of 0.1 or 1% was added to EA, significantly improving the recovery of BPS. The final protocol included elution with 1% HCOOH in EA, which yielded a better BPS recovery of 79% (Figure S3). Parabens, benzophenones, and naphthols were mainly eluted in the DCM fraction (>90%), while bisphenols were mainly eluted in the EA fraction (>90%) (Figure S4).

### 3.2. Concentrations in FF and Urine Samples

**Figure 1:**
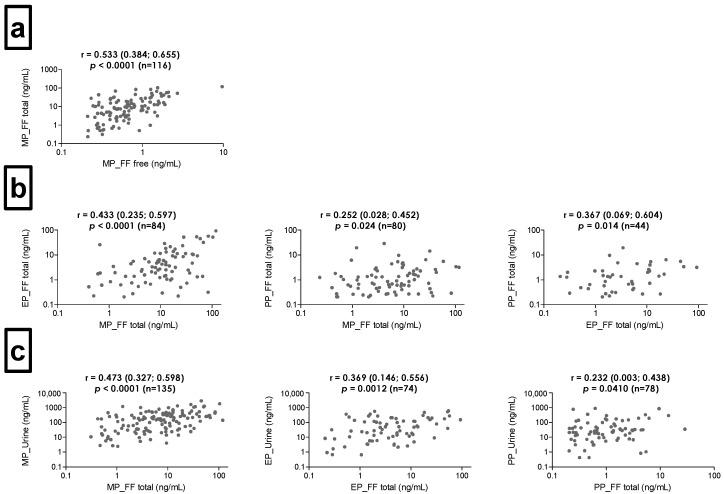
Spearman’s correlation plots for total paraben concentrations in urine and follicular fluid samples. Correlation between (**a**) free and total concentrations of methylparaben in follicular fluid, (**b**) total concentrations of parabens in the follicular fluid and (**c**) between the matrices. Plots include only samples with concentrations >LOD.

A total of 10 out of the 14 substances of interest (MP, EP, PP, BP, BPA, BPS, BP-1, BP-3, 1-NP, 2-NP) were detected in the FF samples, but only three parabens (MP, EP, PP) were detected in more than 50% of the samples analyzed after enzymatic hydrolysis (Table 1). MP was present in free form in more than 78% of samples, with a median concentration of the free form (0.474 ng/mL) accounting for only 7.6% of the total concentration (6.18 ng/mL), and a moderate but significant correlation of 0.533 (*p* < 0.0001) was noted between these concentrations (Figure 1a). Of the remaining substances, eight (BP, BPAF, BPB, BPE, BPF, BPS, BP-1, 1-NP) were not found in free form, and for five (MP, EP, PP, BPA, BP-3, 2-NP), the detection frequency ranged from 3.1 to 34.4%. Of the bisphenols, only BPA was detected in the FF samples. Its detection frequency was <50% for the total concentration and <20% for the free form. Similar results (detection rates) were obtained for both cases and controls (Table 1 and Table S3). A statistically significant but rather weak correlation (r < 0.5) was observed between paraben concentrations in FF samples. MP and EP concentrations showed the strongest correlation (Figure 1b).

A total of 12 out of the 14 compounds analyzed were detected in urine samples (MP, EP, PP, BP, BPA, BPE, BPF, BPS, BP-1, BP-3, 1-NP, 2-NP), eight of which were quantified in more than 50% of samples (MP, EP, PP, BP, BPA, BP-1, BP-3, 2-NP) (Table 1). MP was most frequently determined (98.6%) at the highest concentrations (median: 149 ng/mL) among parabens, while BP was least frequently detected (73.3%) at the lowest concentrations (median: 0.878 ng/mL). Benzophenones were detected with a similar frequency, in almost 90% of the samples, but the median concentration of BP-3 (5.10 ng/mL) was higher than that of BP-1 (2.72 ng/mL). Also, BPA was measured in more than 80% of the samples, while its substitutes, BPF and BPS, were measured in only 19.9% and 10.3% of the samples, respectively. The total concentrations of parabens in the FF samples were significantly correlated with their concentrations in urine samples (*p* < 0.05); however, the correlation was weak for all three parabens (r < 0.5) (Figure 1c, Table S4).

**Figure 2:**
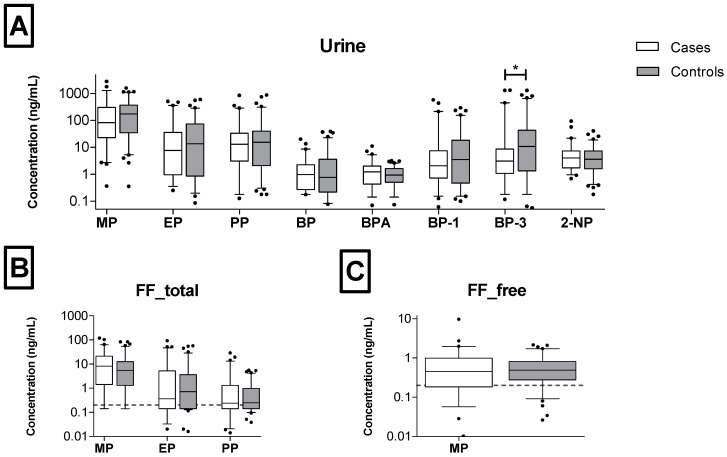
Box plots of (**A**) total urinary phenolic concentrations and (**B**) total and (**C**) free concentrations of parabens in follicular fluid. Analytes with detection >50% were only plotted. Box plots’ description: line—median, box—25th and 75th percentile, whiskers—5th and 95th percentile, dots—results below 5th and above 95th percentile. Dotted line—LOD in FF samples (0.2 ng/mL). *—*p* < 0.05 (Mann–Whitney U test).

The distribution of concentrations in the case and control groups is displayed in Figure 2. Only for BP-3 did the concentrations in urine samples differ significantly (Mann–Whitney U test, *p* = 0.0396), with the median concentration in the control group being higher (13.5 ng/mL) than in the case group (4.11 ng/mL). For other substances, the measured concentrations were similar for both groups in both the FF (*p* = 0.169–0.839) and urine (*p* = 0.127–0.884) samples (Table 1).

## 4. Discussion

According to the World Health Organization, one in six people experience difficulties related to infertility during their lifetime [30]. Infertility is not exclusive and can affect any group of people. Despite a solid understanding of the issue, more research is still required to determine the risk factors/etiological factors. Infertility can result from physiological factors that affect both women and men, but in 10–20% of cases, the root cause of infertility remains unknown [31]. Therefore, the impact of environmental factors, such as exposure to synthetic chemicals, is becoming a topic of interest worldwide. Of particular importance are substances capable of disrupting the endocrine system, known as endocrine-disrupting chemicals (EDCs). In this study, our objective was to compare the concentrations of 14 EDCs in urine and FF samples from healthy women (male-diagnosed infertility, control group) and women diagnosed with idiopathic infertility (cases). Idiopathic infertility, as a non-physiological cause, was an inclusion criterion in the case group, considering that chemical exposure was a likely contributing factor.

The FF serves as a complex environment in which the oocyte grows and matures. It is composed of many essential biomolecules, the qualitative and quantitative content of which vary over the course of the follicle development [32]. However, the concentrations of synthetic chemicals during the stimulatory course can also change, as has been observed for perfluorooctanoic acid (PFOA) [33]. The crossing of chemicals through the blood-follicle barrier appears to hinge on their physicochemical properties, particularly lipophilicity. This phenomenon has been observed for PFASs and more lipophilic POPs such as organochlorine compounds (OCs) or polychlorinated biphenyls (PCBs) [20,34,35]. Furthermore, the observed health effect can vary depending on the number of chlorine atoms in the organic molecule [36]. Age has also been identified as a potential factor in the transfer of lipophilic chemicals from the blood to FF [20]. The presence of chemicals in FF can also be attributed to various lifestyle factors, including food consumption and the use of personal care products [23].

It is challenging to definitively assert whether the presence of synthetic chemicals in the FF has a negative impact on female reproductive health. Numerous studies have reported the presence of historical and emerging POPs, which are known for their endocrine activity, but their association with fertilization parameters is inconsistent. Some studies have found no discernible effect between the concentration of PFASs, PCBs and OCs, considering the individual substances and their mixtures, in the FF and parameters related to ovarian reserve or IVF outcomes [21,26,37]. In contrast to these findings, other studies have demonstrated a negative association between the presence of PCBs and PFASs in the FF and ovarian reserve, ovarian response, and embryo quality [20,36]. An association between reduced ovarian reserve and the concentration of non-persistent EDCs in FF has also been observed [25,26,38], with phenols contributing significantly to this effect [26]. Ovarian function can be disrupted by alterations in steroidogenesis, which negatively affect the fertilization process. Both in vitro [39,40,41] and in vivo [42] studies have shown that exposure of the follicle to EDCs can interfere steroid production.

In our study, the total concentrations of three parabens were measured in more than 50% of the FF samples. These results are consistent with data for women from Sweden and Estonia [25], but median concentrations differed between studies. In the mentioned study, the median MP, EP and PP concentrations were 30.7, 0.06 and 6.90 ng/mL, respectively. In our study, the MP and PP levels were lower, at 6.18 and 0.245 ng/mL, respectively, but higher for EP, at 0.575 ng/mL. The concentrations of free forms of parabens were determined in Chinese women, and, likewise, MP, EP and PP were measured in more than 50% of the FF samples [26], while only MP was measured in our study. The median of free MP in both populations was similar and no differences in distribution were observed between controls and cases for both Polish women, 0.427 and 0.441 ng/mL, and Chinese women, 0.361 and 0.439 ng/mL [26]. Interestingly, the authors quantified BPS in 78.7% of the samples and BPA was not detected in any sample. In our study, free BPA was detected in 16.2% of FF samples, while, after enzymatic cleavage, its detection rate rose to 41.8%. BPS was detected only in <1% of samples analyzed after hydrolysis. Since the free form is considered biologically active, unlike phase II metabolites such as glucuronides, their presence in the FF may be of health concern. These findings point to the prime importance of local metabolic and transport mechanisms within the ovary and that these mechanisms may serve to protect developing oocytes against toxic exposures. They also stress the importance of studying phase II metabolites for their potential bioactivity in reproductive toxicology. The identification of certain phase II metabolites in follicular fluid may offer new approaches to the development of biomarkers of exposure and reproductive risk.

Group discrepancies in the distribution of concentrations between FF samples collected from a group of women with normal and reduced ovarian reserve were not observed for phthalates [42] or parabens [26]. On the contrary, the metabolite concentrations of butylhydroxytoluene (BHT, commonly used antioxidant) were higher in women with a diminished ovarian reserved and were negatively associated with markers of ovarian reserve [38]. In our study, although we did not measure any health outcomes, we observed no differences between the concentrations of the substances measured in the FF and urine samples from women in the case and control groups. Only for BP-3 was the distribution in urine samples statistically significant, with higher concentrations in the control group compared to cases (*p* = 0.04). Tian et al. [26], on the other hand, found higher benzophenone-4 levels in the FF samples of women with declined ovarian reserve (*p* = 0.008).

FF sampling is usually performed during the IVF procedure; hence, it tends to involve a limited number of women who can be enrolled in this type of studies. Therefore, it would be beneficial to know the relationship between the concentration of chemicals in FF and other matrices, such as blood or urine. In the case of POPs, a strong correlation was reported between blood and FF levels, indicating the possibility of using a blood sample as a surrogate for oocyte exposure [20,35,43]. On the contrary, the results for phthalate metabolites showed moderate to weak correlations between urine and FF concentrations [44,45]. We also observed rather weak, though statistically significant, correlations between urinary and FF paraben concentrations (Figure 1b). This observation may arise from the characteristics of both substance groups. POPs are chemicals with long biological half-lives and, over many years, their concentration in the body remains stable. The situation is quite different for non-persistent EDCs, such as phthalates or parabens, whose urinary concentrations can differ significantly even within a day [46,47]. Typically, the concentrations of substances with a similar structure, applications, and, consequently, similar sources and exposure pathways for humans are correlated. This means that measuring the concentration of one substance can be a good indicator of exposure to others [20,37,45].

It is also worth mentioning that the oocyte is immersed in FF from the antral phase until ovulation; thus, its contact with synthetic chemicals is limited in time, and the measurement of its concentration in the FF reflects a relatively short period of exposure [44,45]. However, the FF is a highly valuable source of information related to female reproductive health, also allowing for the observation of the variable environmental factors affecting fertility parameters. Therefore, further research is still needed to expand the knowledge on the presence of synthetic chemicals in FF, as well as their impact on IVF outcomes.

## 5. Strengths and Limitations

A key advantage of this study is the inclusion of a case and control group, thereby comparing exposures between the two groups of women. Since the groups were similar in age and education, we eliminated these factors as confounders when assessing exposure. This study also provides information on the form in which chemicals of interest occur in FF. As the dominant form is phase II metabolites, we might expect a lower or no biological activity, although this hypothesis requires confirmation in further studies. A limitation of this study is the relatively small size as well as low diversity of both groups. Due to the complex process of collecting FF samples, the possibility of including more participants in a short period of time (less than one year), as required by the project, was very limited. Therefore, we decided to include only women with higher education, who were expected to have a higher exposure to substances present in personal care products, such as parabens or benzophenones [48,49]. Therefore, the results obtained should not be generalizable to the entire population of women in Poland.

## 6. Conclusions

This study examined the concentration of 14 phenols in urine and FF samples collected from 154 women who were patients at a fertility clinic. The results emphasize that environmental chemicals in the follicular fluid predominantly appear as phase II biotransformation products and poorly correlate with their urinary concentrations. The weak correlation between urinary and follicular fluid concentrations challenges the assumption that urine measurements adequately reflect exposure levels in other body compartments, placing more pressure on the development of targeted biomonitoring strategies.

In both groups of women (cases and controls), no differences were observed between the concentrations of most chemicals in urine and FF samples. This result may suggest no relationship between the level of exposure to non-persistent EDCs and fertilization outcomes. However, to confirm this hypothesis, additional studies monitoring health outcomes should be conducted, which was not the focus of this study.

## Figures and Tables

**Table 1:** Detection frequency (%) and concentrations (ng/mL) of phenols in follicular fluid and urine samples.

	FF_Free			FF_Total			Urine		
	%>LOD	GM (±95% CI)	Median (Min–Max)	%>LOD	GM (±95% CI)	Median (Min–Max)	%>LOD	GM (±95% CI)	Median (Min–Max)
**MP**									
All	78.6	0.435 (0.370–0.512)	0.474 (<LOD–9.71)	93.5	4.72 (3.63–6.14)	6.18 (<LOD–119)	98.6	95.8 (72.6–127)	149 (<LOD–2786)
Cases	71.9	0.427 (0.315–0.577)	0.448 (<LOD–9.71)	92.2	5.23 (3.32–8.25)	8.18 (<LOD–119)	98.3	79.4 (50.2–126)	83.0 (<LOD–2786)
Controls	83.3	0.441 (0.366–0.532)	0.483 (<LOD–2.13)	94.4	4.39 (3.17–6.08)	5.47 (<LOD–84.8)	98.8	109 (76.0–156)	173 (<LOD–1600)
**EP**									
All	29.9	nc	<LOD (<LOD–5.00)	56.5	0.747 (0.535–1.04)	0.575 (<LOD–93.3)	80.1	7.54 (5.07–11.2)	8.84 (<LOD–593)
Cases	24.3	nc	<LOD (<LOD–5.00)	54.7	0.679 (0.384–1.20)	0.364 (<LOD–93.3)	81.4	6.57 (3.66–11.8)	7.66 (<LOD–508)
Controls	34.4	nc	<LOD (<LOD–3.36)	57.8	0.798 (0.525–1.22)	0.712 (<LOD–57.0)	79.3	8.28 (4.76–14.4)	13.7 (<LOD–593)
**PP**									
All	3.2	nc	<LOD (<LOD–0.920)	53.9	0.372 (0.294–0.472)	0.245 (<LOD–28.9)	94.5	9.87 (7.06–13.8)	14.1 (<LOD–885)
Cases	3.1	nc	<LOD (<LOD–0.920)	51.6	0.377 (0.242–0.587)	0.235 (<LOD–28.9)	91.5	9.29 (5.34–16.1)	12.9 (<LOD–844)
Controls	3.3	nc	<LOD (<LOD–0.394)	55.6	0.369 (0.281–0.485)	0.245 (<LOD–5.51)	96.6	10.3 (6.66–15.9)	15.4 (<LOD–885)
**BP**									
All	0.0	nc	nc (nc)	12.3	nc	<LOD (<LOD–7.26)	73.3	0.961 (0.741–1.25)	0.878 (<LOD–38.7)
Cases	0.0	nc	nc (nc)	6.3	nc	<LOD (<LOD–4.47)	76.3	0.946 (0.659–1.36)	0.980 (<LOD–20.1)
Controls	0.0	nc	nc (nc)	16.7	nc	<LOD (<LOD–7.26)	71.3	0.971 (0.670–1.41)	0.768 (<LOD–38.7)
**BPA**									
All	16.2	nc	nc (<LOD–1.54)	48.1	nc	<LOD (<LOD–8.12)	86.3	0.839 (0.718–0.991)	0.972 (<LOD–11.0)
Cases	18.8	nc	nc (<LOD–1.54)	46.9	nc	<LOD (<LOD–2.36)	81.0	0.891 (0.665–1.19)	1.21 (<LOD–11.0)
Controls	14.4	nc	nc (<LOD–1.12)	48.9	nc	<LOD (<LOD–8.12)	90.0	0.805 (0.673–0.962)	0.927 (<LOD–3.16)
**BP-1**									
All	0.0	nc	nc (nc)	13.0	nc	<LOD (<LOD–2.69)	83.6	2.88 (2.04–4.05)	2.72 (<LOD–580)
Cases	0.0	nc	nc (nc)	14.1	nc	<LOD (<LOD–1.96)	86.4	2.48 (1.49–4.11)	2.04 (<LOD–580)
Controls	0.0	nc	nc (nc)	12.2	nc	<LOD (<LOD–2.69)	81.6	3.19 (1.98–5.12)	3.50 (<LOD–295)
**BP-3**									
All	9.7	nc	<LOD (<LOD–1.64)	26.6	nc	<LOD (<LOD–9.05)	89.7	6.12 (4.12–9.09)	5.10 (<LOD–1336)
Cases	7.8	nc	<LOD (<LOD–0.283)	23.4	nc	<LOD (<LOD–2.26)	89.8	3.95 (2.31–6.74)	3.08 (<LOD–1336)
Controls	11.1	nc	<LOD (<LOD–164)	28.9	nc	<LOD (<LOD–9.05)	89.7	8.23 (4.69–14.5)	10.7 (<LOD–1307)
**2-NP** All	3.2	nc	<LOD (<LOD–0.530)	46.8	nc	<LOD (<LOD–2.62)	99.3	3.53 (2.97–4.19)	3.66 (<LOD–94.7)
Cases	3.1	nc	<LOD (<LOD–0.233)	37.5	nc	<LOD (<LOD–1.25)	100	3.90 (3.01–5.06)	3.99 (0.688–94.7)
Controls	3.3	nc	<LOD (<LOD–0.530)	53.3	0.071 (0.042–0.118)	<LOD (<LOD–2.62)	98.9	3.30 (2.61–4.18)	3.58 (<LOD–40.8)

LOD—limit of detection, GM—geometric mean, CI—confidence interval, nc—not calculated due to low detection frequency (<50%).

## Data Availability

The original contributions presented in this study are included in the article/Supplementary Material. Further inquiries can be directed to the corresponding author.

## Supplementary Materials

The following supporting information can be downloaded at: https://www.mdpi.com/article/10.3390/jox15010017/s1, Table S1: Analytical and internal standards; Table S2: Concentrations (ng/mL) and precision (%) of QC in spiked follicular fluid samples; Table S3: Detection frequency (%>LOD) in follicular and urine samples; Table S4: Spearman’s correlation coefficient between total concentrations of parabens in urine and follicular fluid samples. Figure S1: Chromatogram GC-MS (TIC) of the blank follicular fluid sample after SPE protocol: (A) elution performed with ethyl acetate; (B) elution performed in two fractions as follows: dichloromethane (B1) and ethyl acetate (B2). The black rectangle indicates the bisphenol A interference area; Figure S2: Tentative identification of bisphenol A interference. MS spectrum of follicular fluid sample (top) and NIST library 2.0 reference spectrum (bottom). Match ratio: >80%; Figure S3: Bisphenol S recovery in follicular samples eluted with ethyl acetate (1), 0.1% HCOOH in ethyl acetate (2), 1% HCOOH in ethyl acetate (3). Follicular fluid spiked with bisphenol S after SPE (response 100%) (4); Figure S4: SPE elution profiles of analytes of interest in follicular fluid samples with dichloromethane (DCM) and 1% HCOOH in ethyl acetate (EA).
