# Supplementary material for: Distribution of Environmental Phenols into Follicular Fluid and Urine of Women Attending Infertility Clinic
Source: J Xenobiot. 2025 Jan 21;15(1):17. doi: 10.3390/jox15010017 (PMC11856404; doi:10.3390/jox15010017)
Supplement: Supplementary file 1 [file jox-15-00017-s001.zip › jox-3337272-supplementary.pdf]

SUPPLEMENTARY MATERIALS

SUBMISSION TO THE JOURNAL OF XENOBIOTICS

Distribution of environmental phenols into follicular fluid and urine of women attending infertility clinic

Anna Klimowska [1], Joanna Jurewicz [2], Michał Radwan [3,4], Paweł Pol [6], Paweł Radwan [5,6], Bartosz Wielgomas [1,*]

Text S1 Analytical and internal standards

The following analytical and internal standards: 2-naphthol (2-NP), benzophenone 1 (BP-1), benzophenone 3 (BP-3), bisphenol A (BPA), bisphenol E (BPE), bisphenol F (BPF), bisphenol S (BPS), butylparaben (BP), ethylparaben (EP), methylparaben (MP), propylparaben (PP), benzophenone 3-d_5 (BP3-d_5, phenyl-d_5) and bisphenol A d_{16} (BPA-d_{16}) were purchased from Sigma-Aldrich (Darmstadt, Germany), while 1-naphthol (1-NP) was bought from Riedel-de Häen (Seelze, Germany). The analytical standards of bisphenol AF (BPAF) and labeled analogs of 1-NP (1-NP-d_7), 2-NP (2-NP-d_7), BPAF (BPAF-d_4), BPF (BPF-d_{10}), BPS (BPS-d_8), and MP (MP-d_4, ring-d_4) were obtained from Toronto Research Chemicals (Toronto, Canada). Standard solutions of butylparaben-$^{13}C_6$ (BP-$^{13}C_6$, ring-$^{13}C_6$, 1 mg mL^{-1} in MeOH) and propylparaben-$^{13}C_6$ (PP-$^{13}C_6$, ring-$^{13}C_6$, 1 mg mL^{-1} in MeOH) were purchased from LGC Standards (Łomianki, Poland).

Table S1 Concentrations (ng/mL) and precision (%) of QC in spiked urine samples

	**LOD** *ng/mL*	**1.0 ng/mL** *ng/mL (%)*	**4.0 ng/mL** *ng/mL (%)*	**12.0 ng/mL** *ng/mL (%)*	**30.0 ng/mL** *ng/mL (%)*	**200 ng/mL** *ng/mL (%)*
**MP**	0.5	2.01 (19.7)	4.89 (19.8)	11.7 (19.9)	27.9 (19.7)	193 (18.4)
**EP**	0.5	0.941 (25.7)	3.83 (12.5)	9.45 (16.2)	25.6 (10.4)	202 (7.4)
**PP**	0.25	0.714 (18.0)	3.44 (26.0)	11.4 (22.5)	31.2 (15.1)	202 (10.7)
**BP**	0.25	0.749 (25.7)	2.93 (25.4)	10.7 (20.8)	30.9 (14.4)	–
**BPA**	0.25	1.48 (14.4)	4.57 (14.3)	10.4 (9.5)	26.4 (8.4)	–
**BPAF**	0.1	0.860 (10.6)	3.80 (7.1)	11.7 (4.6)	31.8 (3.7)	–
**BPB**	0.1	1.04 (10.6)	4.04 (16.7)	12.5 (16.3)	27.1 (11.9)	–
**BPE**	0.1	0.895 (10.0)	3.91 (7.6)	10.7 (11.2)	29.8 (9.7)	–
**BPF**	0.25	0.881 (10.0)	3.73 (14.4)	9.77 (9.2)	25.9 (7.5)	–
**BPS**	0.25	0.824 (13.8)	3.07 (14.8)	9.11 (13.5)	24.1 (7.3)	–
**BP-1**	0.25	1.05 (20.3)	3.75 (8.9)	10.8 (6.8)	29.3 (4.8)	205 (7.0)
**BP-3**	0.25	1.89 (11.1)	4.99 (9.8)	13.1 (11.2)	31.4 (11.6)	215 (4.0)
**1-NP**	0.25	0.851 (9.2)	3.98 (18.2)	11.1 (12.2)	34.5 (8.8)	–
**2-NP**	0.25	1.34 (6.5)	3.91 (8.0)	11.2 (10.2)	27.7 (7.8)	–

Table S2 Concentrations (ng/mL) and precision (%) of QC in spiked follicular fluid samples

	**LOD** *ng/mL*	**0.5 ng/mL** *ng/mL (%)*	**1.0 ng/mL** *ng/mL (%)*	**5.0 ng/mL** *ng/mL (%)*
**MP**	0.2	0.560 (23.1)	0.946 (16.8)	3.86 (9.9)
**EP**	0.2	0.410 (25.6)	0.729 (11.6)	4.47 (13.2)
**PP**	0.2	0.406 (20.0)	0.800 (16.8)	4.60 (14.8)
**BP**	0.2	0.471 (28.4)	1.12 (21.9)	4.62 (10.7)
**BPA**	0.2	0.480 (16.2)	0.914 (26.9)	4.65 (15.3)
**BPAF**	0.2	0.418 (26.6)	0.974 (15.5)	5.06 (5.7)
**BPB**	0.2	0.592 (11.4)	1.03 (13.2)	5.01 (12.9)
**BPE**	0.2	0.522 (16.7)	0.894 (9.0)	5.33 (14.5)
**BPF**	0.2	0.536 (13.9)	0.930 (24.1)	5.32 (13.5)
**BPS**	0.2	0.475 (23.7)	0.949 (14.7)	4.88 (11.3)
**BP-1**	0.2	0.497 (22.6)	0.987 (10.9)	5.72 (5.2)
**BP-3**	0.1	0.443 (13.5)	0.881 (9.5)	4.27 (3.8)
**1-NP**	0.1	0.281 (19.5)	0.587 (24.5)	3.48 (9.4)
**2-NP**	0.2	0.549 (19.6)	1.19 (11.6)	6.18 (6.8)

Table S3 Detection frequency (%>LOD) in follicular and urine samples

	**FF_free**			**FF_total**			**Urine**		
	All	*Subjects*	*Controls*	*All*	*Subjects*	*Controls*	*All*	*Subjects*	*Controls*
**MP**	78.6	71.9	83.3	93.5	92.2	94.4	98.6	98.3	98.8
**EP**	29.9	24.3	34.4	56.5	54.7	57.8	80.1	81.4	79.3
**PP**	3.2	3.1	3.3	53.9	51.6	55.6	94.5	91.5	96.6
**BP**	0.0	0.0	0.0	12.3	6.3	16.7	73.3	76.3	71.3
**BPA**	16.2	18.8	14.4	48.1	46.9	48.9	86.3	81.0	90.0
**BPAF**	0.0	0.0	0.0	0.0	0.0	0.0	0.0	0.0	0.0
**BPB**	0.0	0.0	0.0	0.0	0.0	0.0	0.0	0.0	0.0
**BPE**	0.0	0.0	0.0	0.0	0.0	0.0	4.8	5.1	4.6
**BPF**	0.0	0.0	0.0	0.0	0.0	0.0	19.9	18.6	20.7
**BPS**	0.0	0.0	0.0	0.6	0.0	1.1	10.3	10.2	10.3
**BP-1**	0.0	0.0	0.0	13.0	14.1	12.2	83.6	86.4	81.6
**BP-3**	9.7	7.8	11.1	26.6	23.4	28.9	89.7	89.8	89.7
**1-NP**	0.0	0.0	0.0	26.0	20.3	30.0	47.9	45.8	49.4
**2-NP**	3.2	3.1	3.3	46.8	37.5	53.3	99.3	100	98.9

FF_free – concentration of free form
FF_total – total concentration (sum of free form and II phase metabolites)

Table S4 Spearman correlation coefficient between total concentrations of parabens in urine and follicular fluid samples.

	R (+/- 95% CI)		
	All	*Subjects*	*Controls*
**MP**	0.555 (0.427-0.661) ***	0.634 (0.445-0.769) ***	0.536 (0.361-0.675) ***
**EP**	0.546 (0.417-0.654) ***	0.466 (0.231-0.650) *	0.580 (0.416-0.708) ***
**PP**	0.474 (0.333-0.594) ***	0.553 (0.339-0.712) ***	0.418 (0.221-0.582) ***

R – Spearman correlation coefficient
CI – confidence interval
* p < 0.05; *** p < 0.001

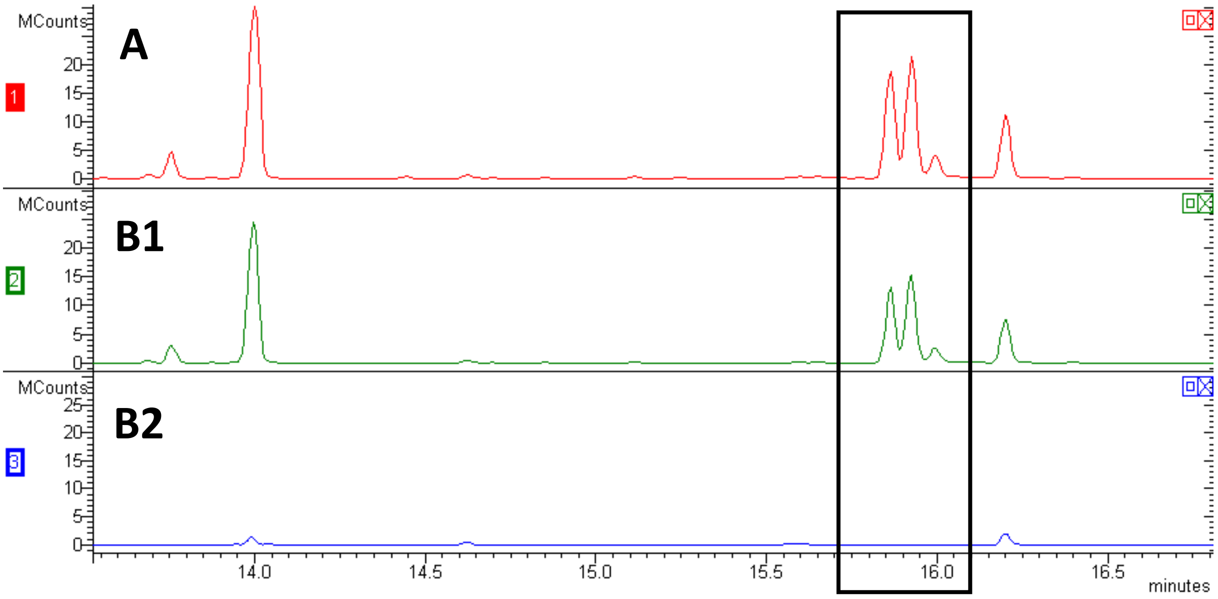

Figure S1 Chromatogram GC-MS (TIC) of the blank follicular fluid sample after SPE protocol: (A) elution performed with ethyl acetate; (B) elution performed in two fractions as follows: dichloromethane (B1) and ethyl acetate (B2). The black rectangle indicates the bisphenol A interference area.

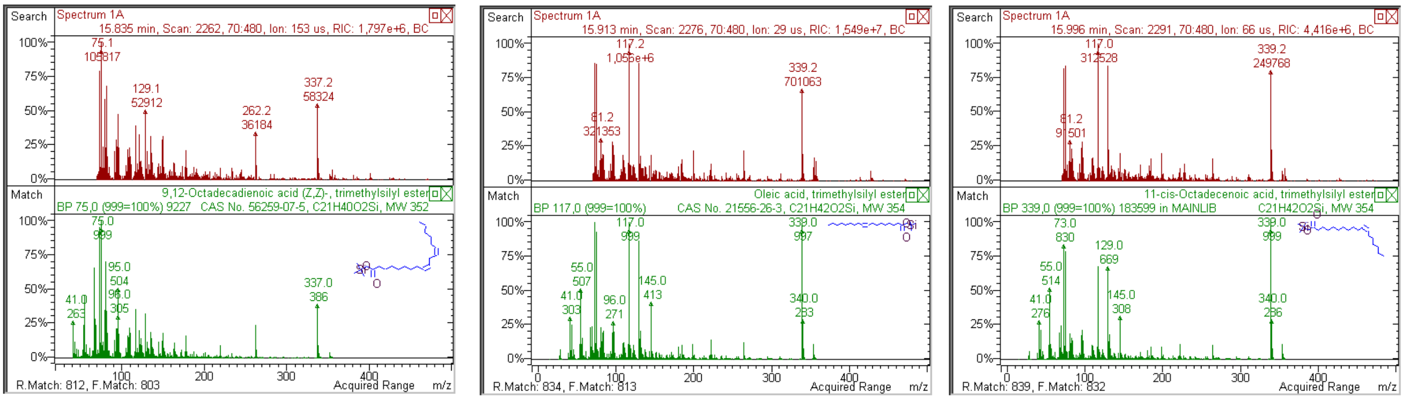

Figure S2 Tentative identification of bisphenol A interference. MS spectrum of follicular fluid sample (top) and NIST library 2.0 reference spectrum (bottom). Match ratio: >80%.

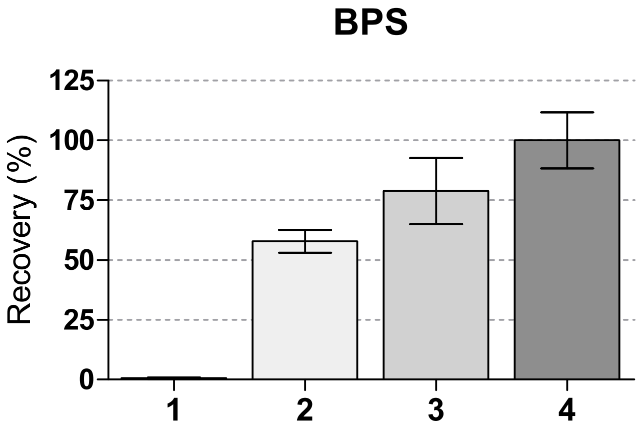

Figure S3 Bisphenol S recovery in follicular samples eluted with ethyl acetate (1), 0.1% HCOOH in ethyl acetate (2), 1% HCOOH in ethyl acetate (3). Follicular fluid spiked with bisphenol S after SPE (response 100%) (4).

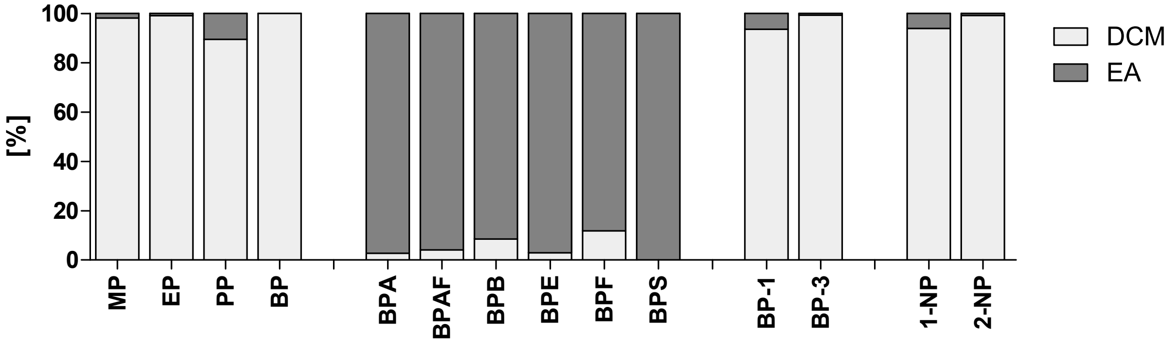

Figure S4 SPE elution profiles of analytes of interest in follicular fluid samples with dichloromethane (DCM) and 1% HCOOH in ethyl acetate (EA).
